# Identification of SSR markers closely linked to the yellow seed coat color gene in heading Chinese cabbage (*Brassica rapa* L. ssp. *pekinensis*)

**DOI:** 10.1242/bio.021592

**Published:** 2017-01-09

**Authors:** Yanjing Ren, Junqing Wu, Jing Zhao, Lingyu Hao, Lugang Zhang

**Affiliations:** State Key Laboratory of Crop Stress Biology for Arid Area, College of Horticulture, Northwest A&F University, Yangling, Shaanxi 712100, People's Republic of China

**Keywords:** *Brassica rapa*, Seed coat color, SSR markers, Genetic map

## Abstract

Research on the yellow-seeded variety of heading Chinese cabbage will aid in broadening its germplasm resources and lay a foundation for AA genome research in *Brassica* crops. Here, an F_2_ segregating population of 1575 individuals was constructed from two inbred lines (brown-seeded ‘92S105’ and yellow-seeded ‘91-125’). This population was used to identify the linkage molecular markers of the yellow seed coat trait using simple sequence repeat (SSR) techniques combined with a bulk segregant analysis (BSA). Of the 144 SSR primer pairs on the A01-A10 chromosomes from the *Brassica* database (http://brassicadb.org/brad/), two pairs located on the A06 chromosome showed polymorphic bands between the bulk DNA pools of eight brown-seeded and eight yellow-seeded F_2_ progeny. Based on the genome sequence, 454 SSR markers were designed to A06 to detect these polymorphic bands and were synthesized. Six SSR markers linked to the seed coat color gene were successfully selected for fine linkage genetic map construction, in which the two closest flanking markers, SSR449a and SSR317, mapped the *Brsc-ye* gene to a 40.2 kb region with distances of 0.07 and 0.06 cM, respectively. The molecular markers obtained in this report will assist in the marker-assisted selection and breeding of yellow-seeded lines in *Brassica rapa* L. and other close species.

## Abstract (precis)

**Summary:** Genetic mapping of the yellow seed coat gene *Brsc-ye* to a 40.2 kb region of the Chinese cabbage (*Brassica rapa* ssp. *pekinensis*) genome will allow marker-assisted selection and breeding of yellow-seeded lines.

## INTRODUCTION

*Brassica rapa* (AA=20), an original parent species of *Brassica napus* (AACC=38) and *Brassica juncea* (AABB=36), is a major rapeseed and vegetable crop worldwide. The seed coat color of *B. rapa* is divided into two major categories, brown/black and yellow; and the qualities of oil and meal from yellow-seeded rapeseed are higher in comparison with those of brown-seeded rapeseed (Stringam et al., 1974). Further, the oil content of yellow seed is 6% higher than that of brown seed because of its significantly thinner seed coat (Stringam et al., 1974; Rashid et al., 1994); and a more transparent oil, lower fiber content and higher protein content are other advantages of yellow seeds (Shirzadegan and Röbbelen, 1985; Getinet et al., 1996). In addition, a lower hull proportion also improves the feed value for poultry and livestock (Tang et al., 1997).

Various reports analyzed the inheritance of seed coat color in *B. rapa*. Mohammad et al. (1942) reported that three independent genes controlled the brown seed coat color, but Stringam (1980) and Rahman et al. (2007) indicated that seed coat color was determined by dominant epistatic gene interactions where the brown color was dominant over the yellow seed coat color. Schwetka (1982) found that the seed coat color difference was determined by one or two genes having epistatic effects. However, Ahmed and Zuberi (1971), Hawk (1982), Chen and Heneen (1992), Zhang et al. (2009) and Xiao et al. (2012) all reported that the brown seed coat color was controlled by a single dominant gene.

Many molecular markers, such as restriction fragment length polymorphism (RFLP), random amplified polymorphic DNA (RAPD), amplified fragment length polymorphism (AFLP), sequence characterized amplified regions (SCAR), simple sequence repeats (SSR) and single nucleotide polymorphisms (SNP) have been used to map genes controlling seed coat color in different *Brassica* materials (Teutonico and Osborn, 1994; Chen et al., 1997; Somers et al., 2001). In *B. rapa*, Rahman et al. (2007) identified one SCAR marker, and 24 SNPs closely linked to a major seed coat color gene. Xiao et al. (2012) obtained the two closest AFLP markers, Y10 and Y6, flanking seed coat color and mapped the gene on chromosome A09. Kebede et al. (2012) concluded that a major quantitative trait loci (QTL; SCA9-2) and a minor QTL (SCA9-1) on A09 and two minor QTLs, SCA3-1 and SCA5-1, on A03 and A05 respectively, were responsible for the variety in seed coat color. Bagheri et al. (2013) also identified a seed coat color QTL (LOD 26) using an F_2_ population.

Because of the existing different genetic patterns for yellow seed coat color, it is necessary to investigate the inheritance models of different yellow seed accessions sources, which will enhance the systemic understanding of yellow seed formation and provide more ways for breeding the yellow seed coat color. Thus, a new yellow-seeded pure line ‘91-125’ of Chinese cabbage (*B. rapa* ssp. *pekinensis*) was investigated in this paper. The objective of this study was to confirm the genetic pattern of seed coat color, and to obtain the closest flanking markers for the marker-assisted selection and breeding of new yellow-seeded *B. rapa* germplasm.

## RESULTS

### Inheritance of seed coat color in Chinese cabbage

An F_2_ population of 1575 individuals, which were planted in 2013 and 2014, was used to analyze the inheritance model of seed coat color. A phenotypic investigation (Table 1) showed that there were 150 brown- and 49 yellow-seeded individuals in 2013, and 1042 brown- and 334 yellow-seeded individuals in 2014. The χ^2^ test indicated that segregation ratios of brown-seeded to yellow-seeded in 2013 and in 2014 were consistent with the expected ratio of 3:1, which showed that the seed coat color trait is controlled by a single gene and that brown is dominant over yellow.

**Table 1.**
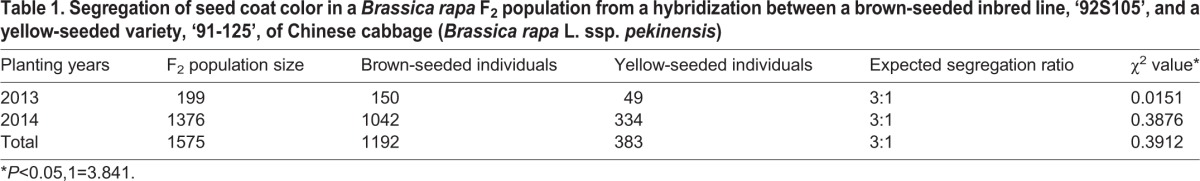
**Segregation of seed coat color in a *Brassica rapa* F_2_ population from a hybridization between a brown-seeded inbred line, ‘92S105’, and a yellow-seeded variety, ‘91-125’, of Chinese cabbage (*Brassica rapa* L. ssp. *pekinensis*)**

### Screening of SSR markers linked to *Brsc-ye* and a preliminary linkage analysis

**Fig. 1.**
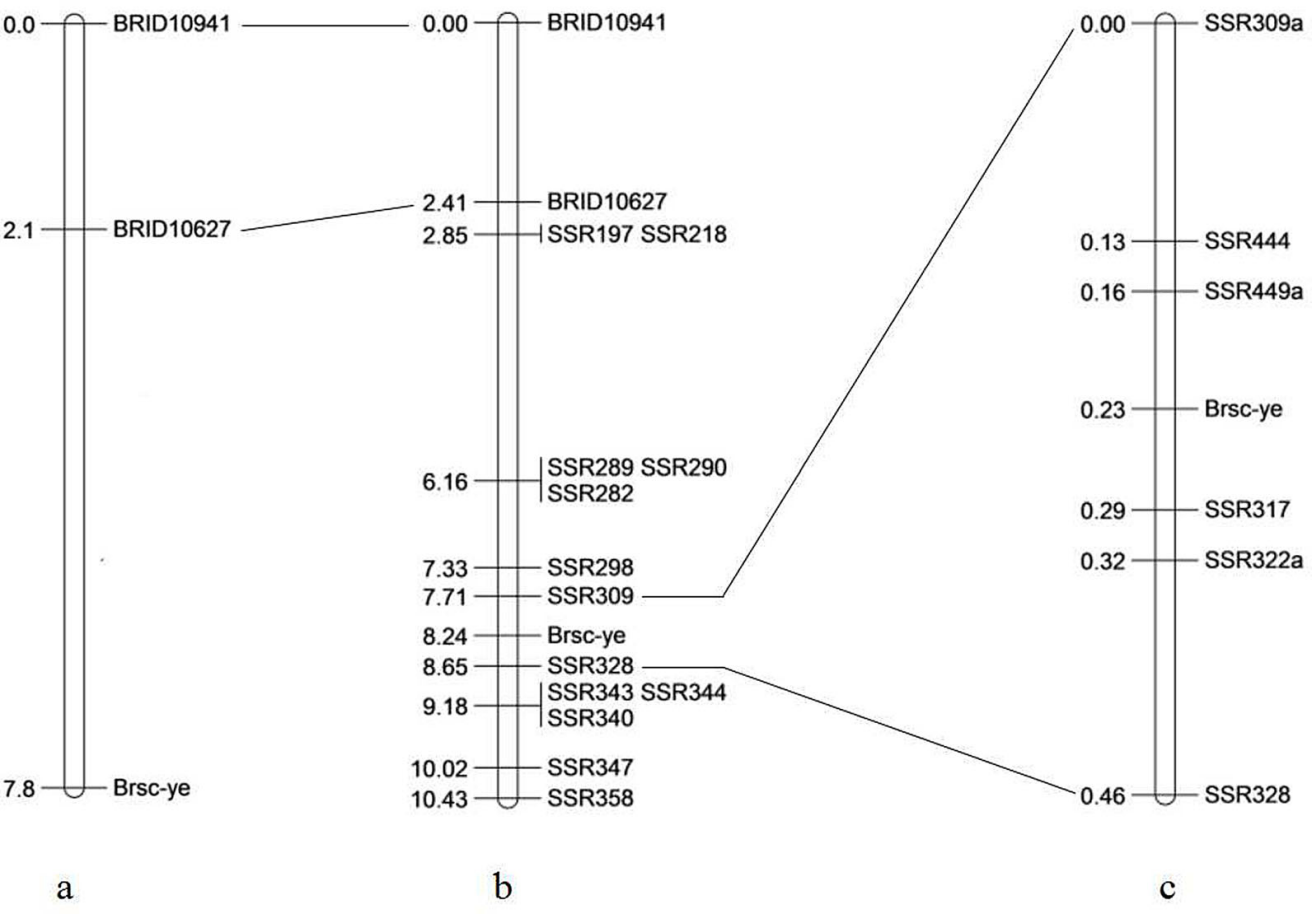
**Genetic maps of the seed coat color gene *Brsc-ye*.** Genetic distances are showed in centiMorgans (cM) on the left side. (a) Genetic map of seed coat color gene *Brsc-ye* using two published SSR markers located on A06. (b) A primary genetic map of the seed coat gene constructed using 15 markers in 199 individuals of the F_2_ population in 2013. (c) Fine genetic map of the seed coat color gene based on 1575 individuals of the F_2_ population.

First, one SSR primer pair, BrID10941, located on A06, which was selected from 120 SSR primer pairs on ten chromosomes published in BRAD database (http://brassicadb.org/brad/), amplified consistent polymorphic bands between the two parents and two DNA bulks. Consequently, 24 additional primer pairs on the A06 of the RCZ16_DH population were synthesized and used to screen for polymorphic bands between the two parents and the two DNA bulks. The other SSR primer pair, BrID10627, was able to amplify polymorphic bands. Thus, these two primer pairs initially mapped the seed coat color gene *Brsc-ye* on chromosome A06, with BrID10627 being closer to the target gene than BrID10941 (Fig. 1a). Then, 400 primer pairs were designed, based on the sequence information downstream of BrID10627, for the continual detection of polymorphic bands between the two DNA bulks. As a result, 13 SSR primer pairs that amplified the polymorphic bands between the two DNA bulks were selected. These SSR primer pairs were used to detect the amplifying polymorphic bands in 199 individuals of the F_2_ population in 2013. Combining the seed coat colors and the polymorphic bands of 199 individuals, a preliminary molecular linkage map of the *Brsc-ye* gene was constructed (Fig. 1b). The two closest molecular markers, SSR309 and SSR328, limited the target gene in 0.94 cM, and SSR309, a dominant marker, was converted into a co-dominant marker, termed SSR309a. Furthermore, 54 new SSR primer pairs between the positions of SSR 309a and SSR328 were designed and screened. Four new SSR primer pairs were designed and amplified polymorphic bands in the 1376 individuals of the F_2_ population in 2014. Thus, a close linkage genetic map was constructed by the combination of phenotypes and the different polymorphic bands of 1575 individuals of the F_2_ population. The two closest markers, SSR449a and SSR317, flanked *Brsc-ye* at 0.07 cM and 0.06 cM, respectively (Fig. 1c). All of the primers used for fine mapping are listed in Table S1.

### Physical mapping of *Brsc-ye*

**Fig. 2.**
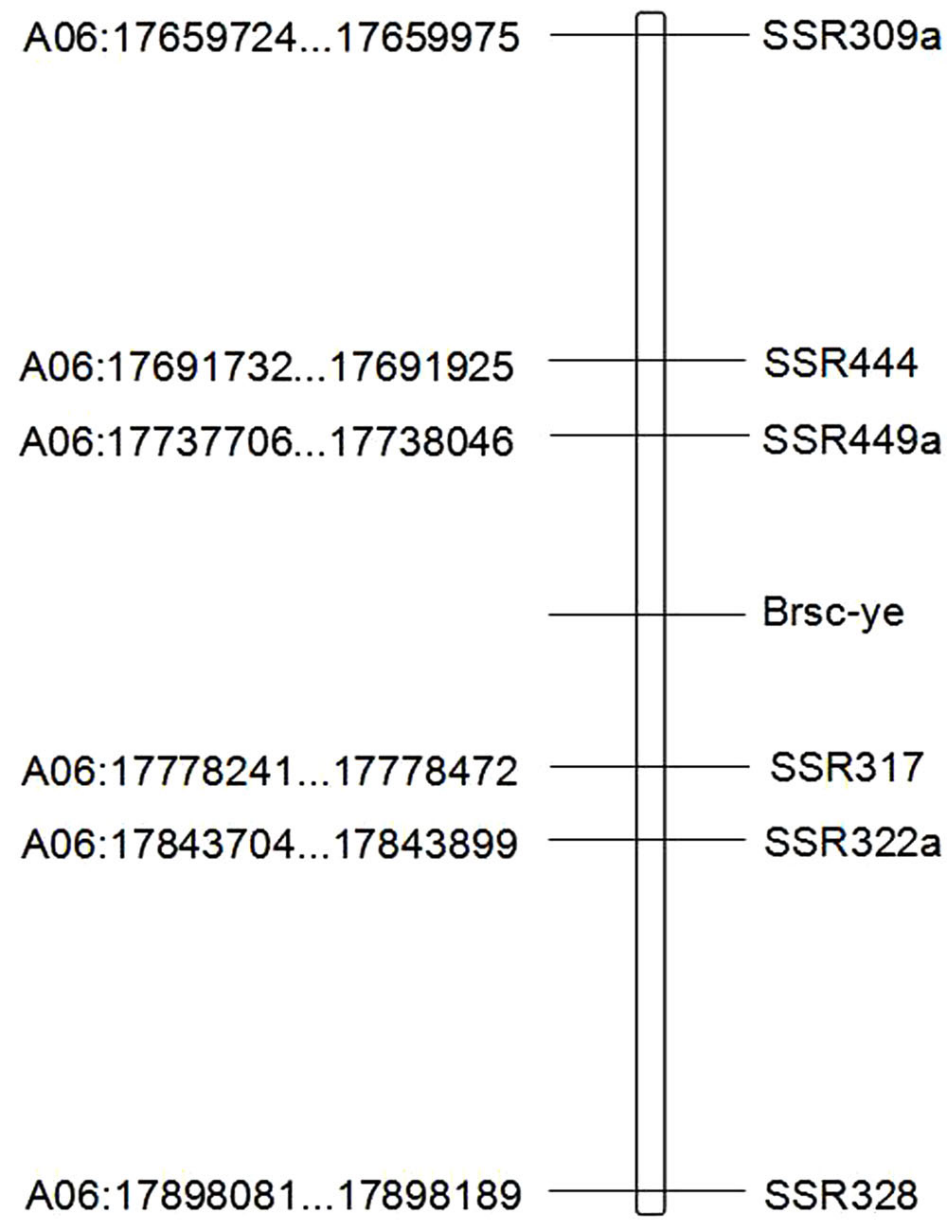
**The physical map of the *Brsc-ye* gene.** Physical positions are shown on the left side, and markers are displayed on the right side.

Combining the data obtained in 2013 and 2014, 1575 F_2_ individuals and six close DNA markers were used to confirm the linkage map of *Brsc-ye.* These six markers mapped the *Brsc-ye* to a region with a genetic distance of 0.46 cM and an average interval of 0.08 cM, in which the two closest flanking markers, SSR449a and SSR317, restricted *Brsc-ye* to an interval of 0.13 cM (Fig. 1c).The physical distance was 40.2 Kb (Fig. 2). The nonconformity between the genetic and physical distances of these markers in this 0.46 cM region, was such that the ratio of the physical interval between two adjacent markers was 1.00:1.44:1.26:2.05:1.70. However, the ratio of the genetic distances between two adjacent markers in the same region was 1.00:0.23:1.00:0.23:1.07. The heterogeneity of genetic and physical distances of these markers revealed that there may be some large chromosome variations between parent materials used in this study and ‘Chiifu-401’ in this 0.46 cM region (Table 2).

**Table 2.**
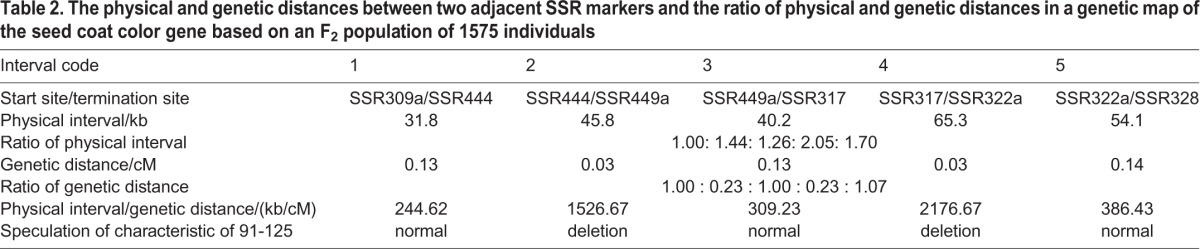
**The physical and genetic distances between two adjacent SSR markers and the ratio of physical and genetic distances in a genetic map of the seed coat color gene based on an F_2_ population of 1575 individuals**

## DISCUSSION

Yellow seed coat is an important agronomic trait for edible oil products in *Brassica* species. However, the main oil crop in *Brassica* species is from *B. napus*, which lacks natural yellow-seeded germplasm resources. Thus, the yellow seed coat gene must be introduced from a close species. The identification and mapping of yellow seed coat gene, as well as associated inheritance studies, are important steps in breeding yellow seed germplasm. The inheritance of yellow seed has been studied for a long time in *Brassica*. Here, the segregation of brown- and yellow-seeded individuals in an F_2_ population of Chinese cabbage showed that the brown seed coat was controlled by one dominant gene, which is in accordance with the results of Ahmed and Zuberi (1971), Hawk (1982) and Chen and Heneen (1992) in *Brassica campestris* (*B. rapa*), but there is no available information on the chromosome locations or molecular markers in their reports. Whether they contain the same gene controlling this trait is unknown. In addition, different inheritance patterns of yellow seed coat in *B. rapa* have also been reported by many researchers. Mohammad et al. (1942) reported that three independent genes controlled the brown seed coat color, and Stringam (1980) indicated that two independent dominant genes were responsible for that trait in turnip rape (*B. campestris*). Rahman et al. (2007) confirmed that the brown seed coat trait was controlled by two independent genes, and that the brown seed coat was dominant in a population of the pure breeding yellow sarson self-compatible variety ‘BARI-6’ from Bangladeshi and a pure breeding brown-seeded self-incompatible variety ‘SPAN’ from Canadian *B. rapa*. Subsequently, Xiao et al. (2012) reported that brown seed coat color was controlled by a single dominant gene and was partially conditioned by the maternal genotype in an oil-used *B. rapa* landrace ‘Dahuang’, originating from the Qinghai-Tibetan plateau. Thus, there are at least four kinds of seed coat color inheritance patterns, which suggest that there are ample variations on yellow-seeded *B. rapa*. Different inheritance patterns of seed coat color hint at different mechanism of seed coat color. New genes or inheritance patterns of yellow seed coat color in *B. rapa* will contribute to *Brassica* cultivar breeding.

Molecular marker technology has been widely used in gene mapping in plants for various interesting traits, such as the *Br-or* gene controlling the orange head of Chinese cabbage (Zhang et al., 2013) and the gene *w* for the white immature fruit color of cucumber (Liu et al., 2015). Close linkage molecular markers and gene location provide preliminary information on the differences and similarities in gene control in different germplasm. Xiao et al. (2012) delimited the *Brsc1* gene on the A09 linkage group (LG) within a 2.8 Mb interval using the two closest AFLP markers, Y10 and Y6. Yuan et al. (2012) found nine QTLs for seed coat color of a summer Chinese cabbage (*Brassica rapa* L. ssp. *pekinensis*) ‘Y195- 93’ (yellow seed) from the variety ‘Xiayang’, which were located on LGs A4, A6, A7 and A10. The most significant QTL, named ScL-2, was located on A6 and explained 80.4% of the phenotypic variance. Kebede et al. (2012) found that one major QTL (SCA9-2) and one minor QTL (SCA9-1) on LG A9, and two minor QTLs, SCA3-1 and SCA5-1, on LG A3 and LG A5, respectively, were responsible for the variation in the seed coat color in a cross between the yellow-seeded cultivar ‘Sampad’ and a yellowish brown seeded inbred line ‘3-0026.027’ of *B. rapa*. The SSR markers from the SCA9-2 region showed a stronger linkage association with seed color compared with the markers from SCA9-1, which suggested that the QTL SCA9-2 is the major determinant of seed color in the A genome of *B. rapa*. Bagheri et al. (2013) identified a seed coat color QTL (LOD26), which co-localized with QTLs for seed size, seed weight, seed oil content, number of siliques and number of seeds per silique using an F_2_ population that was developed by crossing *B. rapa* ssp. parachinensis L58 (‘CaiXin’) with *B. rapa* ssp. trilocularis ‘R-o-18’ (spring oil seed). Because the seed coat color trait is controlled by a single gene and the brown seed is dominant over yellow seed in the present study, as well as in a report by Li et al. (2012), the four published marker-associated primers (Li et al., 2012) were first selected to test whether their polymorphic bands existed in our parental materials. However, the primers did not amplify any polymorphic bands between parents in our study. Thus, there may be various intraspecific differences in seed coat color genes between the materials used in this report and those used in Li et al. (2012)*.* Previous reports (Xiao et al., 2012; Yuan et al., 2012; Kebede et al., 2012; Bagheri et al., 2013) also suggested that the markers used in different papers may be applicable to different yellow-seeded materials.

A nonconformity between the genetic and physical distances on the intervals flanking the target gene *Brsc-ye* locus was found in this study, which suggested that large DNA variations or special DNA sequence construction exists between the parental materials used in this study and ‘Chiifu-401’ sequenced in the BRAD database. Whether the large flanking sequence variations affect *Brsc-ye* will be a new research subject, the re-sequence of Chinese cabbage ‘92S105'and ‘91-125’ may be needed in the future. Tandem arrays were dramatically fractionated after whole genome triplication (WGT) in *B. rapa* (Fang et al., 2012), demonstrating that the genome difference in *B. rapa* is huge. Therefore, it is possible that different yellow-seeded materials show different genetic models due to the expression of different genes, or that the same genetic model may be controlled by different genes. In the future, the cloning and functional verification of candidate gene controlling seed coat color of Chinese cabbage ‘91-125’ will be undertaken. The information obtained in this study will lay a foundation for candidate gene cloning and marker-assisted selection for breeding yellow-seeded *B. rapa* lines.

## MATERIALS AND METHODS

### Plant materials

**Fig. 3.**
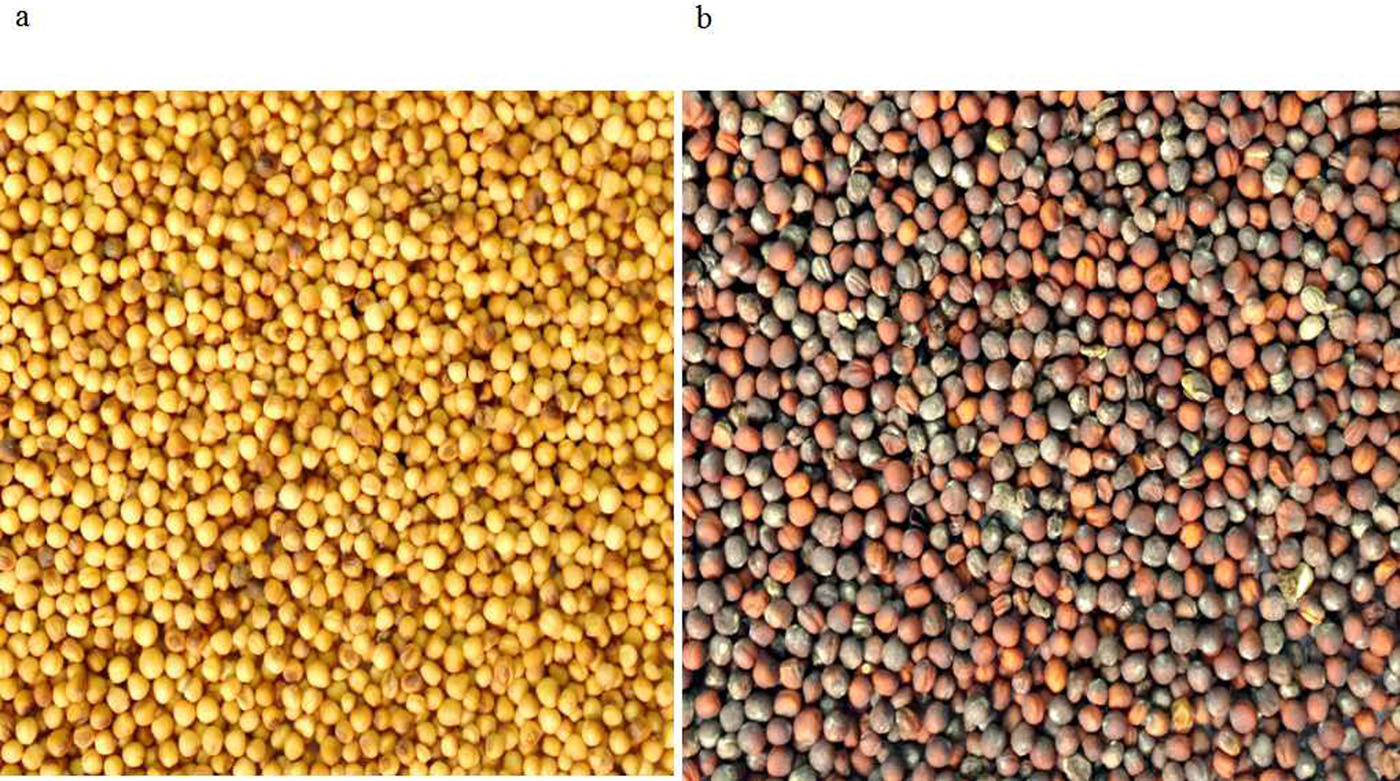
**Seed coat color of parental materials used in this study.** (a) Yellow-seeded parent ‘91-125’. (b) Brown-seeded parent ‘92S105’.

Two Chinese cabbage materials, the yellow-seeded *B. rapa* variety ‘91-125’ (Fig. 3a) and the pure inbred brown-seeded *B. rapa* line ‘92S105’ (Fig. 3b), provided by Chinese cabbage research group of Northwest A&F University, were crossed by artificial emasculation, then an F_2_ population was developed from a single heterozygous F_1_ individual plant. The F_2_ segregation population and two parental lines were planted in the experimental station of Northwest A&F University, Yangling, China, in 2013 and 2014. The 1575 individual plants of the F_2_ population were used to map the seed coat color gene. The seed coat colors of individuals in the F_2_ population were divided into brown and yellow types by visual observation at the seed maturation stage.

### DNA extraction

Genomic DNA of 1575 individuals and two parents were extracted from young fresh leaves by the modified CTAB (cetyl trimethyl ammonium bromide) method (Porebski et al., 1997). The quality of DNA was assessed by electrophoresis in a 1.0% agarose gel, and the final DNA concentration was adjusted to 100 ng/μl with double distilled water. Eight DNA samples each of brown-seeded and yellow-seeded individuals were randomly chosen to construct one brown-seeded and one yellow-seeded DNA bulk, respectively, which were used to screen special markers linked to seed coat color.

### SSR primer design and PCR amplification

The 144 published SSR primer pairs in the *Brassica* database (BRAD) from RCZ16_DH population were used for primary selection. Among these 144 published SSR primer pairs, 10 primer pairs targeted A01, A02, A04, A05, A07, A08 and A10. There were 20 primer pairs for A03 and A09, and 34 primer pairs for A06. In addition to these primers, others were designed by SSR Hunter software (Li and Wan, 2005) and Premier 5.0 software (Premier, Canada) based on the *B. rapa* genome sequence (Wang et al., 2011). PCR amplification was performed using two DNA bulks in 20 μl volume reaction solutions containing 1 μl template DNA (100 ng/μl), 1 μl of each primer (10 μmol), 10 μl PCR All-in-One Premix (HEART, Xi'an China) and 7 μl double distilled water in a BioRad S1000 96 Thermal Cycler. The PCR protocol was as follows: initial denaturation at 94°C for 5 min, followed by 29 cycles with denaturation at 94°C for 30 s, annealing at the appropriate temperature for different primer sets for 30 s, extension at 72°C for 30 s, and then a final extension at 72°C for 5 min. The PCR products were separated on 9% or 12% native polyacrylamide gels with 1× TBE buffer at a constant voltage of 240 V for 1.5 h and then visualized by silver staining system.

### Genetic and linkage analysis

Seed coat color was grouped into brown or yellow by visual observation of mature seeds, and the number of brown-seeded and yellow-seeded individuals was counted separately. The inheritance pattern of seed coat color was determined by the segregation ratio of brown-seeded and yellow-seeded individuals according to the Mendelian ratio. The segregation data of the seed coat color trait and the polymorphic markers in the F_2_ population were used for mapping the gene using JoinMap 4.0 software. The LOD score of 6.0 was used for map construction. The recombination values were converted into genetic map distances (cM) using the Kosambi mapping function (Kosambi, 1943).
